# Anti-inflammatory effects of *Lactococcus lactis* NCDO 2118 during the remission period of chemically induced colitis

**DOI:** 10.1186/1757-4749-6-33

**Published:** 2014-07-29

**Authors:** Tessalia Diniz Luerce, Ana Cristina Gomes-Santos, Clarissa Santos Rocha, Thais Garcias Moreira, Déborah Nogueira Cruz, Luísa Lemos, Adna Luciana Sousa, Vanessa Bastos Pereira, Marcela de Azevedo, Kátia Moraes, Denise Carmona Cara, Jean Guy LeBlanc, Vasco Azevedo, Ana Maria Caetano Faria, Anderson Miyoshi

**Affiliations:** 1 Departamento de Biologia Geral, Instituto de Ciências Biológicas, Universidade Federal de Minas Gerais, Av. Antônio Carlos, 6627 – 31270-901 Belo Horizonte, MG, Brazil; 2 Departamento de Bioquímica e Imunologia, Instituto de Ciências Biológicas, Universidade Federal de Minas Gerais, Belo Horizonte, MG, Brazil; 3 Departamento de Ciência de Alimentos, Faculdade de Farmácia, Belo Horizonte, MG, Brazil; 4 Departamento de Morfologia, Instituto de Ciências Biológicas, Universidade Federal de Minas Gerais, Belo Horizonte, MG, Brazil; 5 Centro de Referencia para Lactobacilos (CERELA-CONICET), San Miguel de Tucumán, Argentina

**Keywords:** *Lactococcus lactis*, Colitis, Cytokines, Regulatory T cells, Probiotics

## Abstract

**Background:**

Many probiotic bacteria have been described as promising tools for the treatment and prevention of inflammatory bowel diseases (IBDs). Most of these bacteria are lactic acid bacteria, which are part of the healthy human microbiota. However, little is known about the effects of transient bacteria present in normal diets, including *Lactococcus lactis*.

**Methods:**

In the present study, we analysed the immunomodulatory effects of three *L. lactis* strains *in vitro* using intestinal epithelial cells. *L. lactis* NCDO 2118 was administered for 4 days to C57BL/6 mice during the remission period of colitis induced by dextran sodium sulphate (DSS).

**Results:**

Only one strain, *L. lactis* NCDO 2118, was able to reduce IL-1β-induced IL-8 secretion in Caco-2 cells, suggesting a potential anti-inflammatory effect. Oral treatment using *L. lactis* NCDO 2118 resulted in a milder form of recurrent colitis than that observed in control diseased mice. This protective effect was not attributable to changes in secretory IgA (sIgA); however, NCDO 2118 administration was associated with an early increase in IL-6 production and sustained IL-10 production in colonic tissue. Mice fed *L. lactis* NCDO 2118 had an increased number of regulatory CD4^+^ T cells (Tregs) bearing surface TGF-β in its latent form (Latency-associated peptide-LAP) in the mesenteric lymph nodes and spleen.

**Conclusions:**

Here, we identified a new probiotic strain with a potential role in the treatment of IBD, and we elucidated some of the mechanisms underlying its anti-inflammatory effect.

## Background

Inflammatory bowel diseases (IBDs), such as ulcerative colitis (UC) and Crohn’s disease (CD), have very complex causes that involve genetic, environmental and geographic factors [1]. They are thought to result from inappropriate and ongoing activation of the mucosal immune cells driven by the presence of an abnormal gut microbiota, resulting in chronic inflammation of the gastrointestinal tract (GIT) [2]. It has been shown that infiltrating T lymphocytes responsive to the gut microbiota are associated with a loss of tolerance in the intestinal mucosa [3].

Current IBD treatments include anti-inflammatory drugs, which induce or maintain remission, but are not curative. Moreover, their use is accompanied by several side effects such as allergic reactions, chills, fever, urticaria and liver problems [4,5]. In this context, biologic agents, such as probiotics with anti-inflammatory properties, have been proposed as tools for both the prevention and treatment of IBD [6].

Most of the probiotics used and studied today belong to the lactic acid bacteria (LAB) group and are mainly composed of lactobacilli, which have been isolated from the human GIT. However, probiotics may also include some *Bifidobacterium*[7] and *Streptococcus* strains [8]. Members of the *Lactobacillus* genus have therapeutic properties, such as improvement of the normal microbiota [9,10], prevention of infectious diseases and food allergies [11-13], stabilisation of the gut mucosal barrier [14,15] and modulation of innate and adaptive immune responses [16-20].

On the other hand, the *Lactococcus* genus has received little attention with respect to its probiotic activities, mainly because these bacteria are not usually considered to be commensal [21]. However, *Lactococcus lactis* strains are in constant transit through the GIT after ingestion of fermented dairy and vegetable products, and a few studies have shown that they can exert beneficial effects [20]. Among these, Nishitani *et al.*[22] demonstrated that *L. lactis* subsp. *cremoris* FC possess potent anti-inflammatory activity. Oral administration of *L. lactis* FC reduced inflammatory cytokine production as well as inducible nitric oxide expression in dextran sulphate sodium (DSS)-induced colitis in mice, suggesting that orally administered *L. lactis* FC may have a beneficial impact in human IBD [22]. Thus, the objective of this study was to evaluate the potential mechanisms involved in the anti-inflammatory effects of *L. lactis* strains that are still poorly understood.

## Results

### Anti-inflammatory effect of *L. lactis* on intestinal epithelial cells (IECs) is strain-dependent

**Figure 1:**
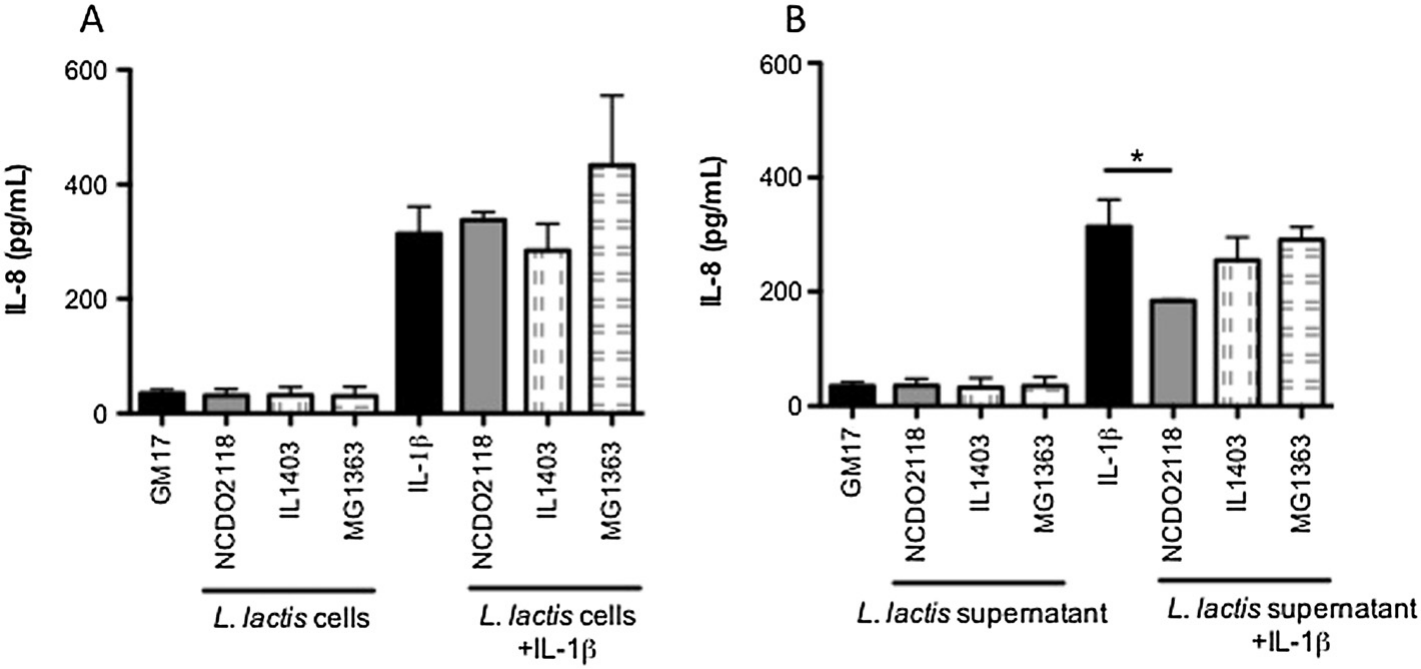
**IL-8 levels after co-incubation of *L. lactis *strains with Caco-2 cells. (A)***L. lactis* cells. **(B)***L. lactis* supernatant. Dash, without addition of IL-1β or bacteria; IL-1β, only IL-1β was added; GM17, only the medium was added. Bars represent the mean and the MSE of three independent experiments. *, p < 0.05.

None of the tested *L. lactis* strains induced IL-8 secretion above background levels, indicating that they do not induce inflammatory events in IECs (Figure 1A and B). To investigate whether *L. lactis* has an anti-inflammatory effect on IECs, the ability of the strains to block IL-8 secretion induced by IL-1β was analysed. Caco-2 cells secreted baseline levels of IL-8, which increased after stimulation with IL-1β. None of the live cell fractions were able to reduce IL-1β-induced IL-8 secretion (Figure 1A); however, the supernatant of NCDO 2118 cultures reduced the production of IL-8 by 45% (Figure 1B), whereas the other 2 supernatants did not show similar effects. Thus, the anti-inflammatory role of *L. lactis in vitro* is strain-dependent.

### Oral administration of *L. lactis* NCDO 2118 alleviates colitis symptoms

**Figure 2:**
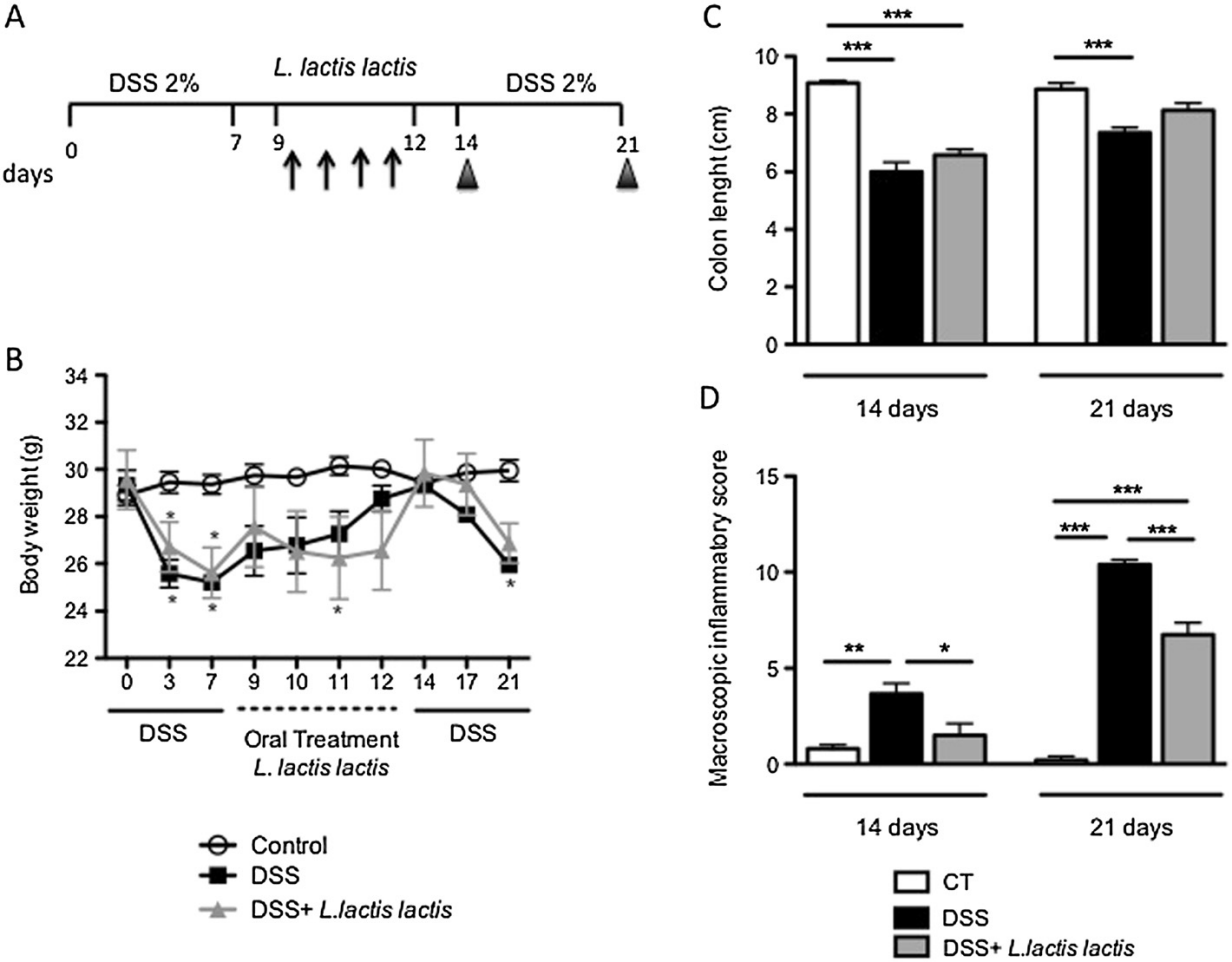
**Oral administration of *L. lactis *NCDO 2118 improved colon shortening and macroscopic score of colitis*. *(A)** Experimental protocol. C57BL/6 mice received 2% DSS for 7 days. *L. lactis* NCDO 2118 was continually administered for 4 consecutive days during the remission period of colitis (arrows) between the first and second course of colitis. The control group received medium. Mice were sacrificed at days 14 and 21 (arrowheads). **(B)** Body weight from day 0 to day 21. **(C)** Colon length measured in cm. **(D)** Macroscopic score of colitis, including scores related to body weight, diarrhea and rectal bleeding. Bars are the mean of 6 mice/group, and the data are representative of three independent experiments; ANOVA, Tukey post-test. *, p < 0.05, **, p < 0.01, ***, p < 0.001.

Based on our *in vitro* results, *L. lactis* NCDO 2118 was then selected for testing *in vivo*. The effect of oral administration of this strain was tested in a murine model of chemically induced colitis during the remission period and after a second colitis cycle. This experimental protocol mimics the remission and active periods of IBD. As shown in Figure 2B, the body weight of mice significantly decreased during DSS treatment compared to the body weight of water-treated mice (control group). After DSS withdrawal, the mice gradually recovered their body weight in all experimental groups. Treatment with *L. lactis* NCDO 2118 did not contribute to a significant change in weight gain (Figure 2B). A reduction in colon length at day 14 in the DSS and DSS + NCDO2118 groups was also observed (Figure 2C). Nevertheless, at the end of the experiment on day 21, oral treatment with *L. lactis* led to the restoration of colon length (Figure 2C). Mice consuming *L. lactis* exhibited significantly reduced clinical symptoms (macroscopic inflammatory score) in the recovery phase and upon colitis induction (Figure 2D), despite the severity of inflammation after the second colitis cycle. These findings suggest that *L. lactis* NCDO2118 administered *in vivo* has an anti-inflammatory effect.

### *L. lactis* NCDO 2118 prevents intestinal inflammation

**Figure 3:**
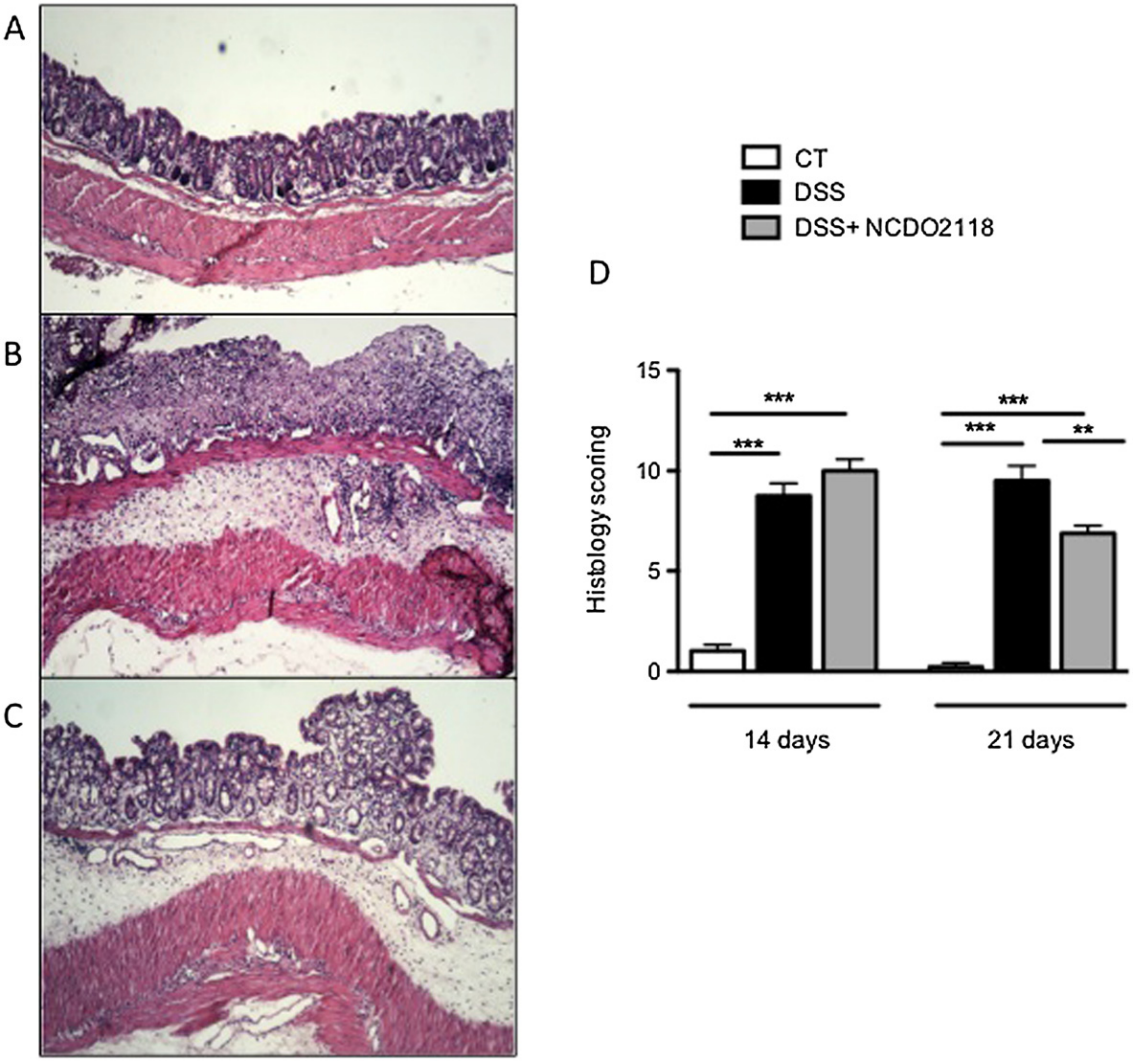
**Oral administration of *L. lactis *NCDO 2118 prevented histological damage induced by colitis*.*** Photograph (X100) of H&E-stained paraffin sections of a representative colon from control **(A)**, DSS **(B)** and DSS + NCDO2118 **(C)** groups at day 21. **(D)** Histological scores of colon sections of DSS-colitis mice with or without oral administration of *L. lactis.* Values represent the means ± MSE (n = 6). **, p < 0.01, ***, p < 0.001.

The ability of *L. lactis* NCDO 2118 to prevent DSS-induced colonic damage was evaluated at the histological level. Colon sections from mice of the control group had an intact epithelium, a well-defined crypt length, and no neutrophil infiltration in the mucosal and submucosal layers (Figure 3A). In contrast, colon tissues from DSS-treated mice showed severe inflammatory lesions throughout the mucosa and submucosa (Figure 3B). Oral administration of *L. lactis* NCDO 2118 ameliorated the histological damage after the second colitis cycle but did not immediately improve the inflammatory status of the gut mucosa on day 14 (Figure 3C, D).

### L. lactis *NCDO 2118 did not alter secretory IgA production*

**Figure 4:**
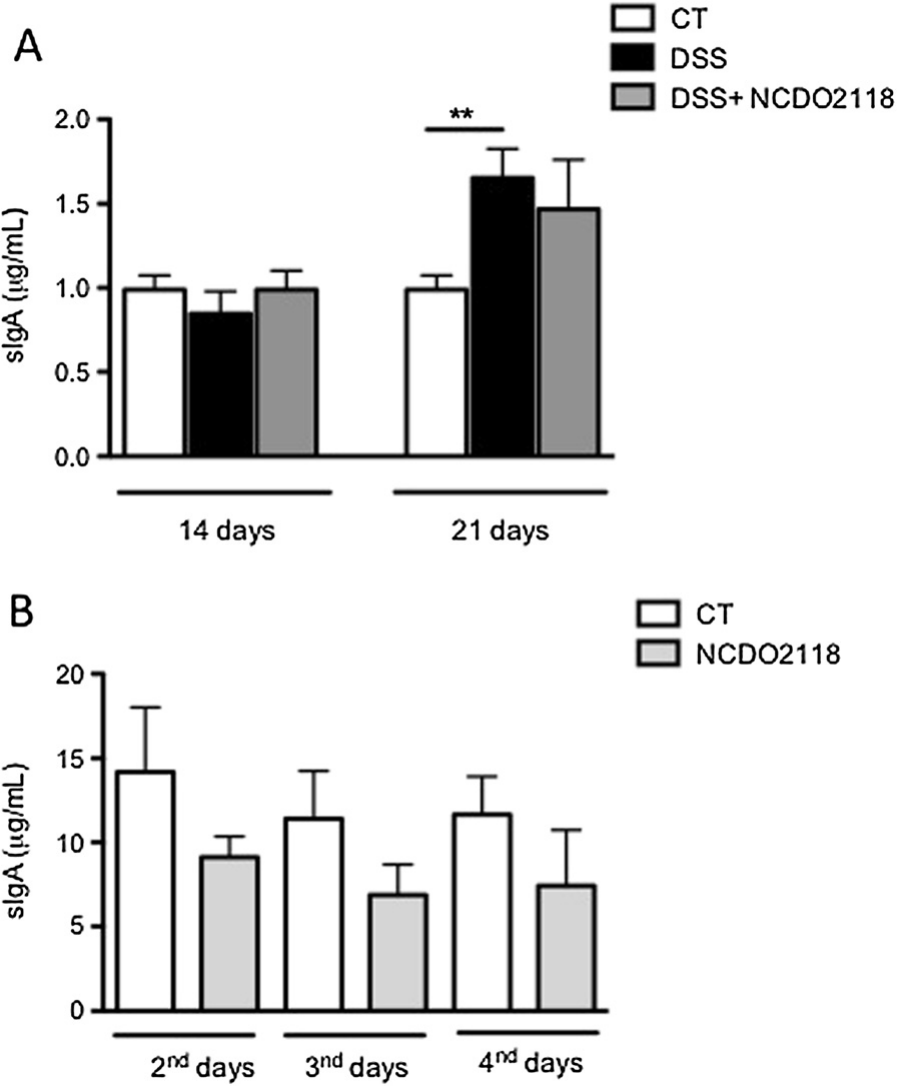
**Oral administration of *L. lactis *NCDO 2118 did not alter secretory IgA production. (A)** Intestinal faeces were collected and total sIgA was measured by ELISA in mice from control, DSS and DSS + NCDO2118 groups. **(B)** Intestinal faeces from healthy mice were collected after 2, 3 or 4 days of *L. lactis* administration, and total sIgA was measured by ELISA. Bars represent the mean ± MSE of 5 mice per group. **, p < 0.01.

Secretory IgA was evaluated in mouse faeces at days 14 and 21. The levels of sIgA were increased only after the second colitis cycle. Oral administration of *L. lactis* NCDO 2118 maintained sIgA production at intermediate levels (Figure 4A). To verify whether *L. lactis* was able to modify the sIgA levels in a physiological scenario, we measured sIgA levels after 2, 3 or 4 days of oral administration of *L. lactis. L. lactis* NCDO 2118 did not alter sIgA production (Figure 4B), discarding the possibility that IgA modulation might be a regulatory mechanism mediated by *L. lactis*.

### *L. lactis* NCDO 2118 modulates the production of cytokines in intestinal tissue

**Figure 5:**
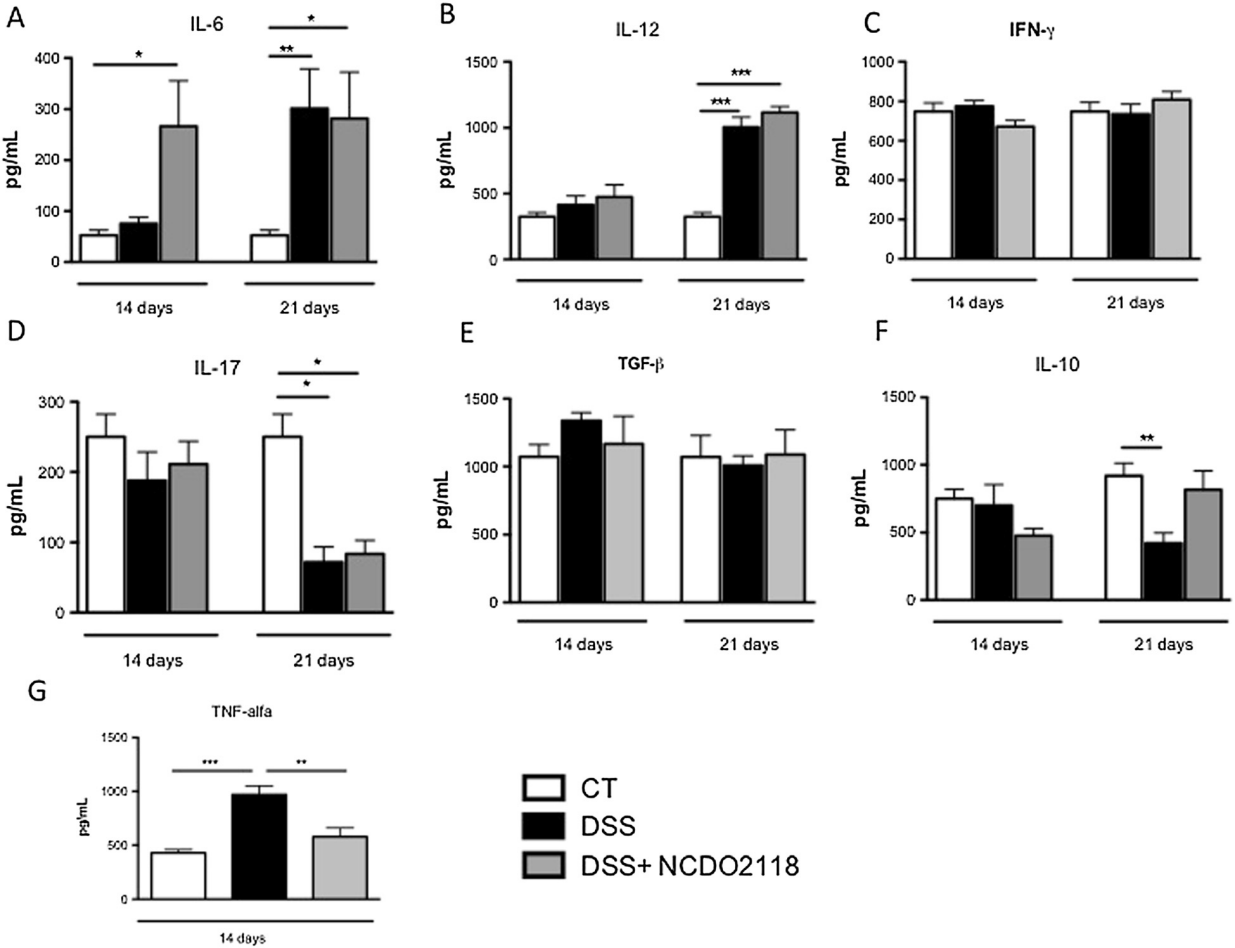
**Effect of *L. lactis *NCDO 2118 on cytokine production by colonic cells.** Colonic IL-6 **(A)**, IL-12 **(B)**, IFN-γ **(C)**, IL-17 **(D)**, TGF-β **(E)**, IL-10 **(F)** and TNF-α **(G)** were measured by ELISA in mice from control, DSS and DSS + NCDO2118 groups. One representative result from two independent repetitions is shown. Bars represent the mean ± MSE of 5 mice per group. *, p < 0.05, **, p < 0.01, ***, p < 0.001.

To further identify potential mechanisms by which *L. lactis* NCDO 2118 exerts its beneficial effects, cytokine profiles in colonic tissue were evaluated at days 14 and 21. Oral administration of NCDO2118 significantly increased the levels of IL-6 at day 14, while the levels of this cytokine were higher at day 21 in both DSS- and DSS + NCDO2118-treated groups (Figure 5A). The exposure of C57BL/6 to 2% DSS led to increased IL-12 levels only at day 21, and *L. lactis* did not affect this phenomenon (Figure 5B). Despite this, the levels of IFN-γ did not change due to DSS or *L. lactis* treatment (Figure 5C). IL-17 levels were reduced at day 21 in both the DSS and DSS + NCDO2118 groups (Figure 5D). TGF-β was not affected by DSS or *L. lactis* (Figure 5E). The anti-inflammatory cytokine IL-10 was significantly decreased in the DSS-treated group but not in the NCDO 2118-treated group at day 21 (Figure 5F). Lastly, the TNF-α level was increased in DSS-treated mice at day 14, while the TNF-α level in the NCDO 2118-treated mice was maintained at a level similar to that of the control group.

### *L. lactis* NCDO 2118 affects cells involved in tolerance

**Figure 6:**
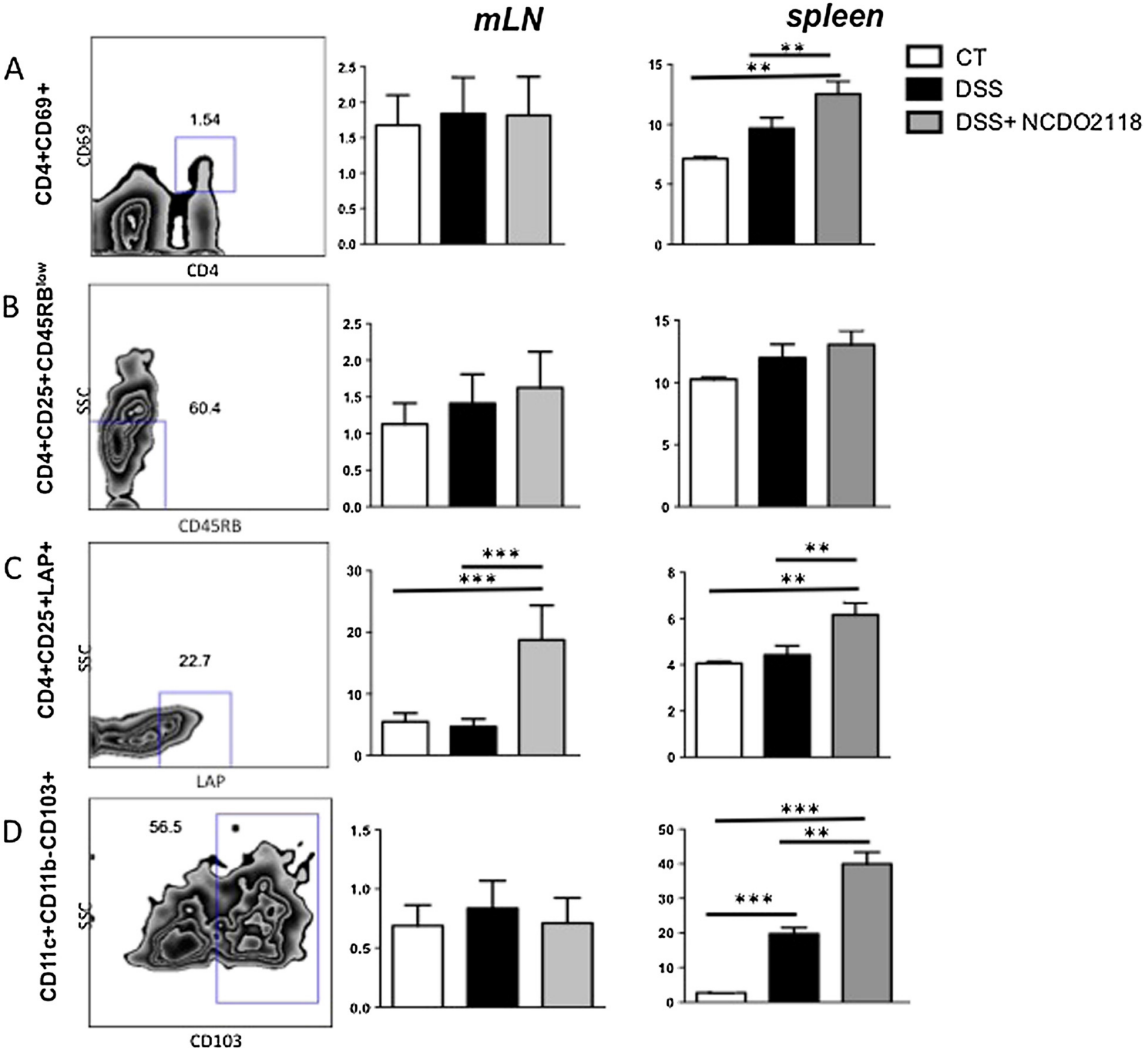
**Effect of *L. lactis *NCDO 2118 on T cells and tolerogenic dendritic cells.** The numbers of activated T cells, regulatory T cells and tolerogenic dendritic cells in chemically induced colitis were determined in the mesenteric lymph nodes (mLNs) and spleen; the cells were stained at day 21. **(A)** Number of CD4 + CD69+ T cells. **(B)** CD4 + CD25 + CD45RB^low^ T cells. **(C)** CD4 + CD25 + LAP + T cells. **(D)** CD11c + CD11b-CD103+ cells. Bars are the mean of 5 mice/group, and the data are representative of two independent experiments; ANOVA, Tukey post-test. **, p < 0.01, ***, p < 0.001.

Because the intestinal inflammation in DSS-induced colitis is triggered by microbial antigens, induction of oral tolerance to microbiota could be one of the potential mechanisms by which *L. lactis* NCDO 2118 stimulates the immune system. Because oral tolerance is maintained mainly by Treg cells [23], we analysed the changes in CD4^+^CD25^+^CD45RB^low^ and CD4^+^CD25^+^LAP^+^ T cells in the mesenteric lymph nodes and spleens of mice. *L. lactis* NCDO 2118 did not alter the numbers of activated T cells in mesenteric lymph nodes. However, this treatment enhanced the number of activated T cells (CD69^+^) in the spleen (Figure 6A), suggesting that some *L. lactis* products are able to activate T cells *in vivo*. The population of CD4^+^CD25^+^CD45RB^low^ regulatory T cells was not affected by DSS or DSS-NCDO2118 treatment (Figure 6B). The same result was observed for CD4^+^Foxp3^+^ Tregs (data not shown). Nevertheless, the levels of CD4^+^CD25^+^LAP^+^ regulatory T cells were increased in the mesenteric lymph nodes and spleens of NCDO 2118-treated mice.

### Increase in the frequency of tolerogenic dendritic cells (DCs) in the group treated with *L. lactis*

The DC population described as tolerogenic alphaE-beta7-(CD103)-expressing DCs was analysed in the mesenteric lymph nodes and spleen. We did not find differences in CD11c^+^CD11b^-^CD103^+^ cells in mesenteric lymph nodes. However, the frequencies of these cells were increased in the spleen during colitis (DSS group), and they were further enhanced in mice of the DSS + NCDO2118 group.

## Discussion

In this study, we showed that culture supernatants of *L. lactis* NCDO 2118 were able to reduce IL-8 production by Caco-2 cells stimulated with IL-1β. Thus, the *L. lactis* NCDO 2118 strain has an immunomodulatory effect in IECs *in vitro*. This conclusion is based on the previous finding that IL-1β triggers transcriptional activation of pro-inflammatory genes in intestinal epithelial cells (IECs), such as IL-8, TNF-α, IL-6, cyclooxygenase-2 (Cox-2) and many others [24,25].

Expression of IL-8 has been shown that this transcriptional factor is overactivated in mucosal cells of IBD patients [26], thus, the search for alternative treatment against IBD, the ability to inhibit the IL-8 secretion or its pathway of production is a good parameter to be considered [27]. Several probiotics, mainly commensals, such as *Lactobacillus rhamnosus* GG [28], *Lactobacillus reuteri*[29], and *Bifidobacterium longum*[29] influence downstream cytokine secretion IL-8 in IECs, while for *L. lactis*, little has been published. Co-cultures of *L. lactis* subsp. *cremoris* FC with Caco-2 cells resulted in significant down-regulation of IL-8 mRNA expression in Caco-2 cells and inhibition of NF-κB nuclear translocation in RAW264.7 cells [22]. Interestingly, we found that IL-8 inhibition is dependent on the strain used for the assay. Similar results were reported by Santos Rocha *et al.*[20]. They also found a strain-dependent immunomodulatory effect by the dairy bacteria *Lactobacillus delbrueckii*[20].

In this study, we showed using an *in vitro* assay that *L. lactis* NCDO 2118 strain has an immunomodulatory effect in IECs. This conclusion is based on the fact that IL-1β triggers transcriptional activation of pro-inflammatory genes in IECs. Thus, IL-1β activates transcription factors, including nuclear factor κB (NF-κB), which induces increased expression of pro-inflammatory mediators, such as IL-8, TNF-α, IL-6, cyclooxygenase-2 (Cox-2) and many others [24,25].

Due to these interesting *in vitro* results, *L. lactis* NCDO 2118 appears to have a potential use as a probiotic for IBD therapy. Thus, *in vivo* experiments were performed to evaluate the effectiveness of this strain in a DSS-induced murine model of colitis.

We have shown that *L. lactis* NCDO 2118 was able to ameliorate a second colitis cycle induced by DSS. In our study, intestinal injury was assessed by a variety of methods, including body weight, colon length and histology. Based on macroscopic and microscopic criteria, *L. lactis* NCDO 2118 inhibited colonic injury. The experimental time period resembles the typical remission period of IBD. We chose to administer the bacteria after the onset of colitis to more closely resemble a clinical scenario, as it is not possible to predict when the disease will start or when it will became active.

*L. lactis* NCDO 2118 also improved the macroscopic symptoms of colitis, especially diarrhea at day 14. However, at this time point, colon length and histological signs were not ameliorated. The analysis of two time points (at day 14 and 21) allowed us to separate two different scenarios in which *L. lactis* exerted effects. We opted to investigate the second scenario because colitis improvement was more evident after the second cycle of colitis.

Several studies have shown that consumption of probiotics is associated with increased gut sIgA levels, which could promote the integrity of the gut immunological barrier by limiting the penetration of bacteria (commensal and pathogenic) into host tissues [30,31]. This is particularly relevant in the DSS model of colitis because DSS is toxic to gut epithelial cells and enhances bacterial translocation [32,33]. However, *L. lactis* did not alter sIgA production after oral treatment.

Cytokines produced in the gut mucosa greatly influence the resulting immunological outcome. The production of anti-inflammatory cytokines induces mucosal tolerance, and high levels of pro-inflammatory cytokines induce a protective immune response and inflammation. The most intriguing aspect of probiotic-induced modulation of immune responses is the effect of probiotics on cytokine production. Few studies have investigated the effect of *L. lactis* NCDO 2118 on cytokine production. Kimoto *et al.*[34] showed that *L. lactis* G50 induces Th1-type immune responses *in vitro*. Pavan *et al.*[35] observed a significant increase in IFN-γ production in the ilea of mice fed *L. lactis* MG1363. In our study, *L. lactis* NCDO 2118 did not alter the production of IFN-γ in colonic tissue.

We found increased levels of IL-6 in the colon of mice fed *L. lactis* NCDO 2118 after colitis induction. In acute situations, such as chemically induced colitis, IL-6-deficient mice had higher levels of inflammation than wild type mice. It appears that IL-6 enhances mucosal repair by epithelial reconstitution [36-40]. Therefore, we speculate that *L. lactis* could promote epithelial repair via IL-6 production resulting ultimately in the prevention of diarrhea as discussed above.

IL-17 is generally thought to have a proinflammatory role in the intestine [41]. However, neutralisation of IL-17 can aggravate acute DSS-induced colitis in mice, suggesting that IL-17 has a protective role in colonic inflammation [42]. In our study, IL-17 levels were diminished in mice that received the second cycle of DSS, but these levels were not affected by oral administration of *L. lactis* NCDO 2118.

IL-10 is most likely the most important cytokine involved in shaping immune responses at the gut mucosa. IL-10-deficient mice spontaneously develop gut inflammation [43]. In the present study, after the second cycle of colitis, IL-10 levels were decreased in the colon of DSS-treated mice, but *L. lactis* NCDO 2118 administration prevented this reduction. Thus, the maintenance of IL-10 levels seems to be responsible, at least in part, for the anti-inflammatory effect of *L. lactis* NCDO 2118.

In the present study, the oral administration of *L. lactis* NCDO 2118 improved the aberrant levels of TNF-α induced by DSS approximately to the control levels. In agreement with this, Nishitani *et al.*[22] demonstrated that *Lactococcus lactis cremoris* drastically reduced the mRNA expression of TNF-α, the major proinflammatory cytokine involved in the DSS-induced colitis model.

To investigate the effect of *L. lactis* on T cell populations, we evaluated these cells in the mesenteric lymph nodes and spleens of mice treated or not with *L. lactis* NCDO 2118 during chronic colitis. IBD is generally believed to be driven by T cells and has been thought to be associated with an increase in inflammatory cytokines, especially from Th1 and Th17 cells. Specialised regulatory T cells counterbalance these proinflammatory responses [44,45]. The numbers of activated T cells expressing the earliest inducible cell surface glycoprotein acquired during lymphoid activation, CD69, were analysed after colitis induction. The numbers of CD4^+^CD69^+^ T cells were increased only in the spleens of mice fed *L. lactis* NCDO 2118. Therefore, some *L. lactis* products were able to activate T cells.

Transfer of naïve CD4+ T cells or innate immune activation in leukopenic mice have been reported to induce colitis, whereas co-transfer of CD4^+^CD25^+^CD45RB^low^ T cells can prevent disease induction [46]. This T cell population was further identified as a regulatory T cell subset. Despite the anti-inflammatory activity of *L. lactis* NCDO 2118, its administration did not enhance the frequency of this regulatory T cell population. However, another type of peripherally induced Treg cells characterised by their surface expression of LAP, which is the N-terminal propeptide of TGF-beta precursor, were increased in the mesenteric lymph nodes and spleens of mice treated with *L. lactis* NCDO 2118. Previously, Di Giacinto *et al.*[17] showed that administration of the probiotic VSL#3 during the remission period of TNBS-induced colitis increases the numbers of regulatory CD4^+^LAP^+^ T cells, and this is essential to the protective effect of the probiotic.

**Figure 7:**
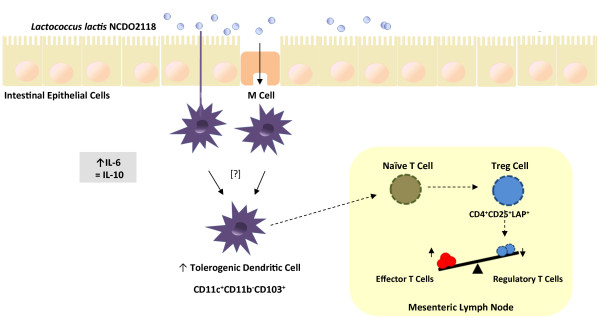
**Schematic model for the immunomodulatory effects of *Lactococcus lactis *NCDO 2118 in DSS-induced colitis.** Orally administered *Lactococcus lactis* NCDO 2118 is able to induce an early increase in IL-6 production and to sustain IL-10 secretion in colonic tissue of the dextran sulphate sodium (DSS)-induced murine model of ulcerative colitis. It also increases the number of local tolerogenic dendritic cells (CD11c^+^CD11b^-^CD103^+^). These cells migrate to the mesenteric lymph nodes and stimulate the expansion of CD4^+^CD25^+^LAP^+^ cells, a regulatory type of T cell (Treg), leading ultimately to downmodulation of colitis.

Because dendritic cells modulate T cell differentiation into effector or regulatory T cells [47], the profile of DCs was evaluated. It has been shown previously that CD103^+^ DCs can induce CD4 + CD25 + Foxp3+ Treg cells in the intestinal mucosa [48]. In the chemically induced colitis model, we found increased numbers of CD11c^+^CD103^+^ DCs in mice treated with DSS. However, oral administration of *L. lactis* NCDO 2118 enhanced the numbers of CD11c^+^CD103^+^ DCs to a greater extent. Gyu Jeon *et al.*[49] recently showed that *Bifidobacterium breve* induced the development of IL-10-producing T cells and that this effect was mediated by CD103^+^ DCs. Thus, *L. lactis* NCDO 2118 may trigger a regulatory phenotype in DCs that drives the expansion of induced regulatory T cells such as CD4^+^LAP^+^. Therefore, we propose a working model for *L. lactis* NCDO 2118 activity *in vivo*, which is depicted in Figure 7.

## Conclusions

In conclusion, we showed that *L. lactis* NCDO 2118 has anti-inflammatory activity in an *in vitro* culture of intestinal epithelial cells (IECs) and in a DSS-induced model of colitis. Moreover, we showed the effectiveness of this *in vitro* screening method for identification of a probiotic strain of *L. lactis* that can be further tested *in vivo.* The mechanisms involved in such anti-inflammatory effects include modulation of colonic cytokines as well as expansion of regulatory T cells and anti-inflammatory DCs. Taken together, our results suggest that not only commensal but also dairy bacteria that are part of our diet can have probiotic effects. The nature of the *L. lactis* NCDO 2118 components that are responsible for its anti-inflammatory effects is under investigation.

## Methods

### Ethics statement

Conventional inbred female C57BL/6 mice, 10 to 12 weeks of age, were obtained from Universidade Federal de Minas Gerais (UFMG), Brazil. Mice were maintained in an environmentally controlled room with 12 h light–dark cycle. All procedures were approved by the local ethics committee for animal research (Comitê de Ética em Experimentação Animal (CETEA) from Universidade Federal de Minas Gerais (UFMG), Brazil- CEUA # 114/2010).

#### *Bacterial strains and growth conditions*

Three *L. lactis* strains were used in this study: *L. lactis* subsp. *lactis* NCDO 2118 [50], *L. lactis* subsp. *lactis* IL1403 [51], and *L. lactis* subsp. *cremoris* MG1363 [52]. They were grown at 30°C in M17 medium (Difco) containing 0.5% glucose (GM17) without agitation or in the same medium solidified with 1.5% agar for 18 hours.

#### *Epithelial cell culture*

Caco-2 cells (ATCC HTB-37), a human colon adenocarcinoma cell line, were cultured in RPMI medium (Sigma) supplemented with 10% (v/v) of fetal bovine serum (FBS) (Gibco), 2 mM L-glutamine, 0.1 mM non-essential amino acids, and 1 mM sodium pyruvate solution in an atmosphere containing 5% CO_2_ at 37°C.

#### *Epithelial cell treatments*

Caco-2 cells were seeded at 3×10^5^ cells/well in 24-well plates and incubated at 37°C with 5% CO_2_ for 24 hours before treatment. Secretion of the pro-inflammatory cytokine IL-8 by the cells was induced by the addition of human recombinant IL-1β (BD Biosciences) to a final concentration of 10 ng/mL. *L. lactis* cultures in the stationary phase of growth were fractionated by centrifugation into supernatant and cells, and each fraction was co-incubated with Caco-2 cells. Bacterial cells were washed 2 times with PBS (137 mM/L NaCl, 2.7 mM/L KCl, 10 mM/L Na_2_HPO4 • 2 H_2_O, 2 mM/L KH2PO4) and added at a *multiplicity* of *infection* (MOI) of 5. The supernatant was filtered at a final concentration of 10% (v/v). Caco-2 cells that were not treated with IL-1β were used as controls. After 6 hours of co-incubation, the supernatant of cell cultures was collected and stored at -80°C until analysis. IL-8 levels were measured using a Human IL-8 ELISA Kit (BD Biosciences) following the manufacturer's instructions. Data from three independent experiments were analysed.

#### *DSS-induced colitis*

Chemical colitis was induced by replacing the drinking water of mice with a 2% (w/v) aqueous solution of dextran sodium sulphate (DSS, MP Biomedicals) for 7 consecutive days. Subsequently, mice received either GM17 medium (DSS group) or *L. lactis* NCDO 2118 (DSS + NCDO2118 group) orally for four consecutive days. Fresh total cultures of NCDO2118 (bacteria plus supernatant at a stationary phase of growth) were prepared daily before being offered to the mice. Because each mouse drank approximately 5 mL of culture per day (data not shown), the total dose of bacteria per mouse was estimated to be 5×10^9^ bacteria/day. Mice were sacrificed either at day 14 (immediately following the oral treatment) or after a second DSS cycle (at day 21). The control groups of mice received M17 or *L. lactis* alone. Throughout the experimental period, all mice had unlimited access to food. A schematic representation of the experimental procedure is shown in Figure 2A.

#### *Macroscopic and microscopic assessment of colitis*

DSS-induced colitis was assessed macroscopically by scoring three major clinical signs—weight loss, diarrhea, and rectal bleeding—at day 14 and day 21 as described by Cooper *et al.*[53]. Body weight loss was calculated as the difference between the initial and actual weight. Diarrhea was determined by assessing mucus/faecal material adhering to anal fur and was confirmed by the presence or absence of faecal pellet formation and continuous fluid faecal material in the colon. Rectal bleeding was defined as diarrhea containing visible blood and gross rectal bleeding. The three major clinical signs (weight loss, diarrhea, and occult/gross bleeding) were scored separately. The macroscopic score was calculated from the score of the clinical signs using the following formula: (weight loss score) + (diarrhea score) + (rectal bleeding score). After the mice were sacrificed, their spleens, mesenteric lymph nodes and colons were excised. Spleens and mesenteric lymph nodes were used for cell population analysis. Colon samples were fixed in formalin and processed for histological analysis. Hematoxylin-eosin-stained sections were blindly scored based on a previously described semi-quantitative scoring system [54]. The following features were graded: extent of destruction of normal mucosal architecture (0: normal; 1, 2 and 3: mild, moderate and extensive damage, respectively), presence and degree of cellular infiltration (0: normal; 1, 2 and 3: mild, moderate and transmural infiltration, respectively), extent of muscle thickening (0: normal; 1, 2 and 3: mild, moderate and extensive thickening, respectively), presence or absence of crypt abscesses (0: absent; 1: present) and the presence or absence of goblet cell depletion (0: absent; 1: present). Scores for each feature were summed up to a maximum possible score of 11.

#### *Colon tissue preparation and cytokine assay*

Colon samples were weighed and homogenised in PBS containing 0.05% (v/v) Tween-20, 0.1 mM phenylmethylsulphonyl fluoride, 0.1 mM benzethonium chloride, 10 mM EDTA and 20 KIU Aprotinin A using a tissue homogeniser (100 mg tissue/ml buffer) [43]. Suspensions were centrifuged at 12,000 g for 20 min at 4°C, and the supernatants were collected for the cytokine assay. The plates were coated with purified monoclonal antibodies reactive for the cytokines IL-6, IL-12, IFN-γ, IL-17, IL-10, TGF-β and TNF-α (BD-Pharmingen) overnight at 4°C. On the following day, the wells were washed, the supernatants were added and the plate was incubated overnight at 4°C. On the third day, biotinylated monoclonal antibodies against the cytokines were added, and the plates were incubated for 2 hours at room temperature. Colour was developed at room temperature with 100 μl/well of orthophenylenediamine (1 mg/ml) and 0.04% (v/v) H_2_O_2_ substrate in sodium citrate buffer. The reaction was interrupted by the addition of 20 μl/well of 2 N H_2_SO_4_. The absorbance was measured at 492 nm using a microplate reader (BIO-RAD).

#### *Secretory IgA (sIgA) assay*

The levels of sIgA were determined by ELISA. Briefly, 96-well plates (NUNC) were coated with Ig goat anti-mouse UNLB antibody in coating buffer (pH 9.6) overnight at 4°C. The wells were washed and blocked with 200 μl of PBS containing 0.25% casein for 1 h at room temperature. Samples were added to the plates and incubated for 1 h at 37°C. The plates were then washed, peroxidase-streptavidin goat anti-mouse IgA-HRP (Southern Biotechnology) diluted 1:10000 was added, and the plates were incubated for 1 h at 37°C. Colour was developed at room temperature with 100 μl/well of orthophenylenediamine (1 mg/ml) (SIGMA) and 0.04% H_2_O_2_ substrate in sodium citrate buffer. The reaction was interrupted by the addition of 20 μl/well of 2 N H_2_SO_4_. The absorbance was measured at 492 nm using an ELISA microplate reader (Bio-Rad).

#### *Antibodies and FACS analysis*

Fluorescein isothiocyanate-conjugated (FITC) mAbs against CD69, CD25 and CD11c; phycoerythrin (PE)-conjugated mAbs against CD45RB and CD11b; and biotinylated antibodies against CD103 were utilised. Streptavidin-Cy5-Chrome was purchased from BD Biosciences. The biotinylated anti-human LAP (TGF-β) antibody was purchased from R&D Systems. Surface staining was performed according to standard procedures at density of 1×10^6^ cells (isolated from the spleen or mesenteric lymph nodes) per well. The samples were analysed in a FACScan instrument (BD), and the results were analysed by FlowJo Software (Tree Star Inc.).

#### *Statistical analysis*

The results were expressed as the mean ± standard error of the mean (SEM). Normal distribution of the samples was confirmed by the Kolmogorov–Smirnov test. The significance of differences among groups was determined by Student’s t-test or analysis of variance (ANOVA) (Tukey’s post test). Means were considered significantly different when P < 0.05.

## Abbreviations

COX: Cyclooxygenase; CD: Crohn’s disease; DC: Dendritic cell; DSS: Dextran sodium sulphate; GIT: Gastrointestinal tract; IBDs: Inflammatory bowel diseases; IEC: Intestinal epithelial cell; IL: Interleukin; IFN: Interferon; LAB: Lactic acid bacteria; LAP: Latency-associated peptide; NF-κB: Nuclear factor κB; sIgA: Secretory IgA; TGF: Transforming growth factor; TNF: Tumour necrosis factor; Tregs: Regulatory T cells; UC: Ulcerative colitis.

## Competing interests

The authors declare that they have no competing interests.

## Authors’ contributions

Conceived and designed the experiments: ACGS, CSR, AMCF, DCC, JGL, VA, AM. Performed the experiments: TDL, ACGS, CSR, TGM, DNC. Analysed the data: TDL, ACGS, ALS, LL, MA, VBP, KM. Wrote the paper: TDL, ACGS, JGL, AMCF, AM, VA. All authors read and approved the final manuscript.

## Authors’ information

Ana Maria Caetano Faria and Anderson Miyoshi share credit for senior authorship.
